# Case Report of Distal Descending Colonic Torsion in a German Shepherd Dog Highlights Diagnostic Limitations of Survey Radiography

**DOI:** 10.1002/ccr3.72070

**Published:** 2026-02-25

**Authors:** Nicole Moody, Ashley M. Power

**Affiliations:** ^1^ VCA Veterinary Referral & Emergency Center Westbury New York USA

**Keywords:** canine, colon, colopexy, radiographs, torsion

## Abstract

Survey radiographs may be inconclusive in diagnosing colonic torsion, underscoring the importance of clinical suspicion in directing surgical intervention.

## 1 Introduction

Colonic torsion is a fairly uncommon pathologic twisting of the colon about its longitudinal axis [1, 2, 3]. While torsion and volvulus are often used interchangeably in the literature, volvulus is distinct in that it involves rotation of a viscus about its mesenteric axis [1, 2, 3]. Intestinal torsion and volvulus are considered surgical emergencies and can lead to life‐threatening sequelae including bowel wall necrosis and septicemia; therefore, prompt diagnosis and surgical exploration is imperative for a favorable outcome [4, 5, 6, 7]. Although the precise etiology of colonic torsion is unknown, some authors suggest that breed, sex, and pre‐existing gastrointestinal disease may have an influence [6, 7, 8, 9, 10]. Many cases present with non‐specific clinical signs such as vomiting, tenesmus, lethargy, abdominal pain, retching, and diarrhea [4, 7, 8, 10, 11, 12]. Few case studies have described “dynamic torsion,” which can present as more chronic and non‐specific gastrointestinal symptoms, further confounding diagnosis [7, 12].

Historically, the prognosis for dogs with colonic torsion and volvulus was estimated to be poor; however, recent reports indicate more favorable outcomes [5, 9, 13], with the largest study to date (28 dogs) reporting a 92.9% survival to discharge [7]. Radiographic features of colonic torsion had not been well described in the literature until a recent study of 14 dogs, which described segmental distention of the colon, focal narrowing of the colon, displacement of the cecum, displacement of the descending colon, and mild to no small intestinal distention as findings consistent with colonic torsion on plain radiographs [12]. However, in all cases in that study, barium enema was required for definitive diagnosis prior to making the decision to proceed to surgery [12]. While computed tomography (CT) findings have been reported in a small case series of dogs with colonic torsion [4], this modality is often not available after hours, except at some tertiary referral facilities.

Definitive diagnosis of colonic torsion can be elusive due to often vague presenting clinical signs and lack of pathognomonic findings on plain radiographs [7, 12]. Furthermore, there is an overall paucity of literature compared to more common conditions such as gastric dilatation‐volvulus, which may have a similar clinical presentation. The current report aims to highlight the limitations of our current diagnostic capabilities to identify colonic torsion and emphasizes that high clinical suspicion is crucial in prompting timely surgical intervention.

## 2 Signalment and History

A 3 year old, 31.4 kg female spayed German Shepherd dog was presented late in the evening to the emergency service at Veterinary Referral and Emergency Center of Westbury for acute onset of restlessness, vomiting and diarrhea with tenesmus. There had been possible dietary indiscretion (buffalo chicken, blue cheese) the day prior to presentation; however, no known foreign material ingestion. The dog had previously experienced other episodes of gastrointestinal upset consisting of waxing and waning vomiting and diarrhea for 3 months prior to presentation. The dog had been fed a raw diet.

## 3 Presentation and Diagnostics

On presentation, the dog was anxious, pacing, and panting. Heart rate was 120 bpm; temperature could not be obtained initially owing to patient temperament. Physical examination indicated mild ptyalism, moderate abdominal pain, and inability to sit down comfortably.

**FIGURE 1:**
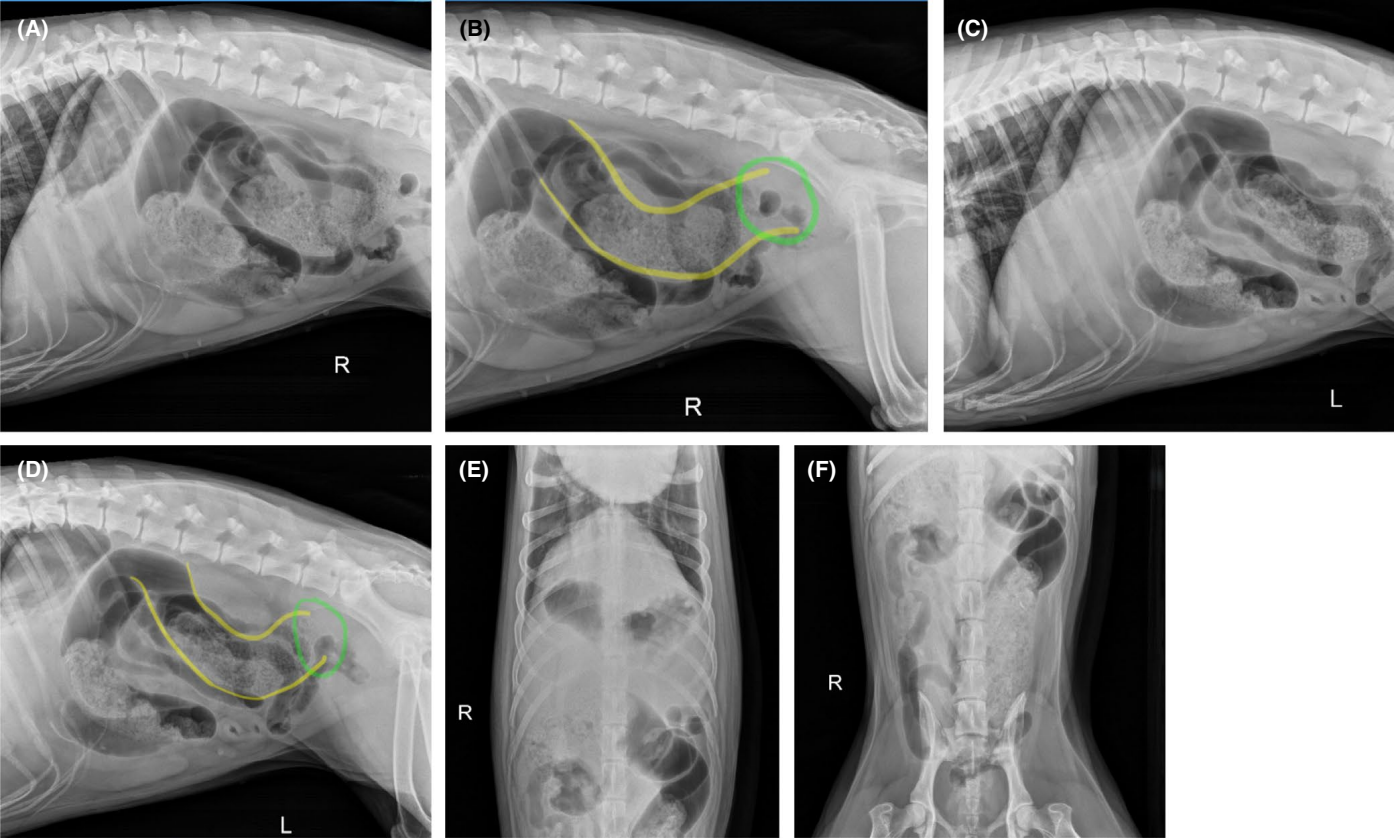
Abdominal radiographs obtained at presentation. Atypical colonic course and distention. (A) Cranial right lateral view. (B) Caudal right lateral view. Yellow lines indicate the coursing of the descending colon, which becomes indistinct in the region indicated by the green circle. (C) Cranial left lateral view. (D) Caudal left lateral view. Yellow lines indicate the coursing of the descending colon, which becomes indistinct in the region indicated by the green circle. (E) Cranial ventrodorsal view. (F) Caudal ventrodorsal view.

Initial treatments included methadone (0.3 mg/kg IV; Breckenridge Pharmaceutical Inc., Berkeley Heights, NJ), dexmedetomidine (5 mcg/kg IV; Zoetis, Parsippany, NJ), maropitant (1 mg/kg IV; Zoetis, Parsippany, NJ), and an intravenous bolus of lactated Ringer's solution (20 mL/kg; Braun Medical Inc., Bethlehem, PA). Following fluid bolus, peripheral lactate via lactometer was normal (1.6 mmol/L; range, < 2.5 mmol/L). Complete blood cell count indicated mild neutrophilia (13.46 × 10^3^μL; range, 3.05–12.1 × 10^3^μL). Serum biochemistry revealed mildly elevated BUN (33.3 mg/dL; range, 9–29 mg/dL) and hypertriglyceridemia (20 mg/dL; range, 30–130 mg/dL). Resting cortisol was normal (2.5 μg/dL, range, 1–5 μg/dL). Abdominal radiographs were obtained and submitted for stat radiologist review; findings included mild to moderate gas distension of the jejunum, atypical and slightly redundant course of the colon, gas‐stippled heterogeneous feces, mineral debris and moderate volume of gas within the colon and reduced serosal detail due to visceral crowding (Figure 1).

The radiologist's consultation suggested ruling out colonic torsion versus colitis with pneumocolonogram, barium colonogram, and/or CT. Retrograde passage of a red rubber catheter was attempted, but was unsuccessful due to apparent encounter of a mechanical obstruction in the distal colon. After‐hours CT was not available at this facility.

## 4 Treatment and Outcome

**FIGURE 2:**
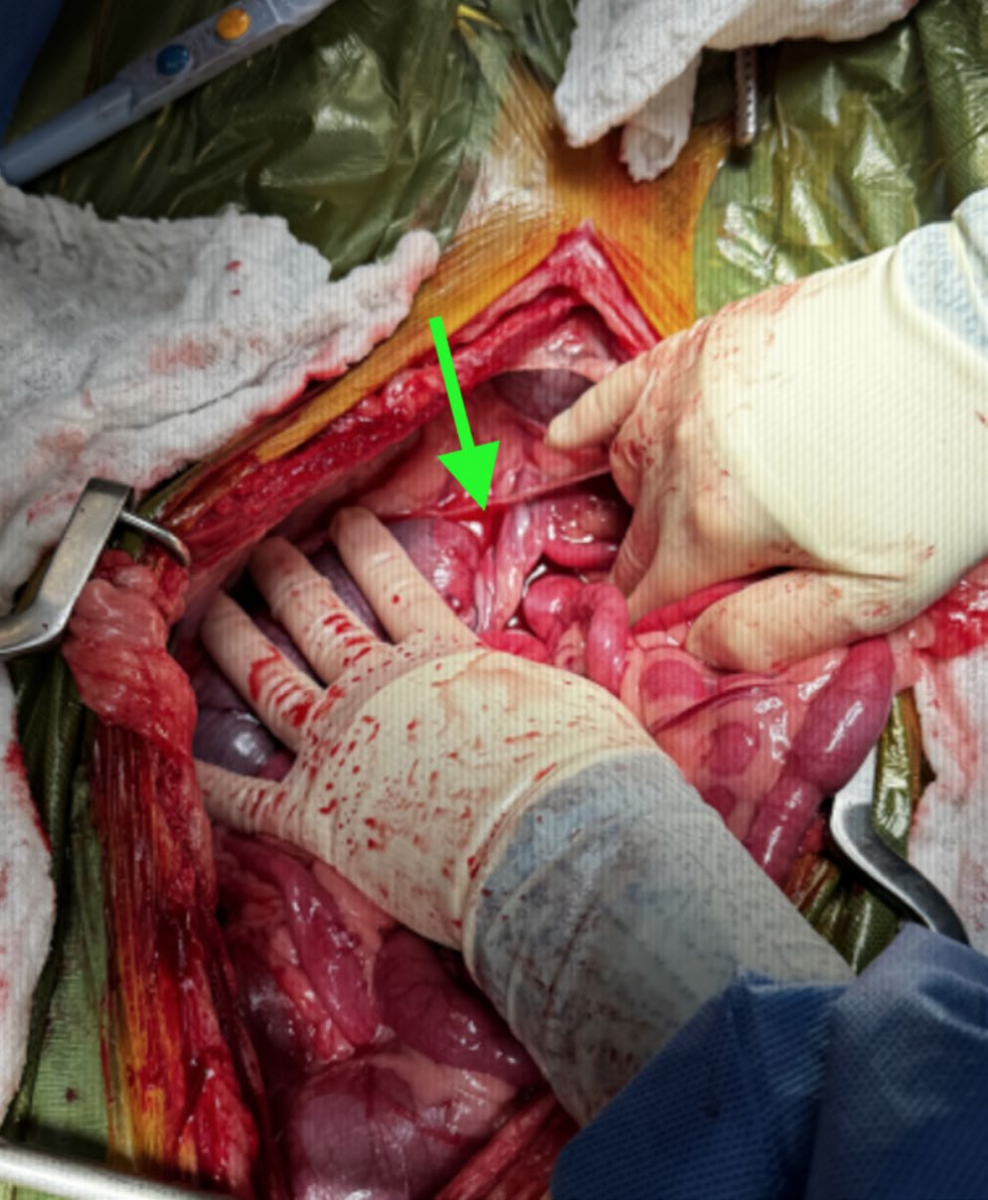
Intra‐operative photograph showing 360 degree distal colonic torsion about the caudal mesenteric artery (arrow). Cranial is toward the bottom of the photograph.

Due to persistent abdominal discomfort and concern for colonic torsion that could not be definitively demonstrated radiographically, the dog underwent emergent exploratory laparotomy. The dog was administered additional methadone (0.1 mg/kg IV) as well as cefazolin (22 mg/kg IV q90 min; Hikma Pharmaceuticals, Berkeley Heights, NJ). Anesthetic induction was performed with propofol (5 mg/kg IV; Zoetis, Parsippany, NJ), and anesthetic maintenance was achieved via inhalant with isoflurane (Dechra, Northwich, England). The dog was positioned in dorsal recumbency, and a ventral midline celiotomy was performed. The abdomen contained scant serosanguineous peritoneal effusion. The descending colon was found to be torsed 360 degrees about the pedicle of the caudal mesenteric artery (Figure 2). There was severe dilation of the colon orad to the torsion, but the bowel appeared healthy with no indication of devitalization. The small bowel, stomach, kidneys, adrenal glands (palpated but not visualized), urinary bladder, liver, and spleen were unremarkable. The colon was de‐rotated back to normal position. The entire bowel was run multiple times to ensure appropriate orientation. A left‐sided colopexy was performed with peritoneal incisions made in two locations. The adjacent sites on the descending colon were scarified with gauze, and the two locations were apposed with cruciate sutures of 3‐0 polypropylene. A gastrocolopexy was performed with a seromuscular incision made along the greater curvature of the stomach, and the adjacent transverse colon was scarified with gauze. These sites were apposed with cruciate sutures of 3‐0 polypropylene. These two sites were approximately 6 cm apart from one another, and each incision was approximately 2 cm in length. Similarly, the body wall incisions for the colopexy of the descending colon were each approximately 2 cm in length. This method was chosen to provide an additional site of fixation to prevent future torsion of the transverse colon. Two sites of fixation were performed for each site to provide additional security against failure should one of the suture lines fail. Finally, a routine right‐sided incisional gastropexy was performed, with paired, approximately 4 cm incisions made over the caudal most rib and adjacent pyloric antrum, apposed with two simple continuous suture lines of 2‐0 polydioxanone. All positioning and orientation of the gastrointestinal tract was confirmed acceptable. The bowel remained viable in appearance during the entirety of the procedure. Routine celiotomy closure was performed. The time from presentation to induction of anesthesia was 3 h.

The dog recovered uneventfully and was kept hospitalized for monitoring, fluid replacement, and pain management. In‐hospital treatments included lactated Ringer's solution (120 mL/kg/day IV), methadone (0.1 mg/kg IV q4), trazodone (150 mg PO q8), and cefazolin (22 mg/kg IV q8). A renal panel was performed the morning after surgery, which indicated normalized BUN (16.5 mg/dL; reference range, 9‐20 mg/dL). The dog continued to have diarrhea in‐hospital; however, it was eating well and comfortable upon abdominal palpation. The dog was discharged approximately 24 h post‐operatively with carprofen (62.5 mg PO q12 × 7 days; Zoetis, Parsippany, NJ) and cefpodoxime (200 mg PO q24 × 14 days; Dechra, Northwich, England).

Following discharge from the hospital, diarrhea resolved within 24 h. The dog was doing well at the time of 2 week suture removal. At the time of this writing (1 month post‐operative), the dog continues to do well without reported gastrointestinal signs.

## 5 Discussion

The current study demonstrates a favorable outcome in a case of distal descending colonic torsion following prompt surgical intervention. Importantly, emergent surgery was pursued in this case despite lack of definitive diagnosis on plain radiographs. As recommended by the literature [12] as well as by the consulting radiologist in this case, passage of a red rubber catheter was attempted to perform either contrast‐ or gas‐enhanced radiography (i.e., barium enema or pneumocolonogram). However, this was unsuccessful in this case owing to the very distal location of the torsion.

Although advanced imaging was another consideration to confirm the diagnosis prior to proceeding with surgery, computed tomography (CT) was not available after hours. This is commonly the case in many emergency hospitals, either due to a lack of staff trained to operate CT equipment during off‐hours or the complete absence of CT machinery at the facility. Furthermore, the only current report in the literature describing CT features of colonic torsion is a small case series limited to 5 dogs; although these authors describe a “whirl sign” and distention of the mesenteric vasculature as highly suggestive of colonic torsion, a pathognomonic feature has not been identified [4].

The current report, therefore, emphasizes the importance of utilizing clinical suspicion in directing a decision to pursue emergent exploratory surgery when colonic torsion is a differential diagnosis, even when it cannot be definitively identified on imaging. In addition to the mentioned technical or logistic shortfalls of imaging capabilities, there is also the consideration of dynamic colonic torsion being fairly common in the populations that have been studied [7, 12]. Indeed, recent studies report that 35.7% [7] and 36% [12] of dogs were found to have a resolution of colonic torsion at the time of surgery, thereby presuming (or speculating) a dynamic component to colonic torsion in these cases. Such features underscore the inherent diagnostic challenges in definitively identifying colonic torsion or volvulus prior to surgery. That said, prognosis for these cases can be grave when not diagnosed or acted upon quickly. This places clinicians in a difficult position, where timely intervention is critical, though definitive diagnosis can remain elusive.

Owners should be counseled on the risk of a negative explore; however, in most cases, these dogs are also at‐risk breeds for gastric dilatation‐volvulus (German shepherd dogs, labrador retrievers, Great Danes) [7, 10, 11, 12, 13], and so may benefit from a prophylactic gastropexy at time of explore, even if colonic torsion is not identified. Therefore, these authors would argue that surgical intervention would not be entirely without benefit in such cases. Ultimately, it is paramount for the clinician to consider breed and conformational predispositions, characteristic (though not necessarily definitive) radiographic features, and the patient's presenting clinical status to assess the likelihood of colonic torsion, and therefore, whether there is sufficient clinical suspicion to prompt emergent surgery. As illustrated by this case, timely decision‐making is critical to minimize the risk of bowel necrosis or septicemia, and to improve patient outcomes.

## 6 Conclusion & Clinical Relevance

This report describes a case of distal descending colonic torsion where survey radiographs were inconclusive, and barium enema/pneumocolonogram were not feasible owing to the distal location of the torsion. This case emphasizes the importance of clinical suspicion in directing timely surgical exploration, which played a key role in achieving a successful outcome. Future consideration should be given to identifying unique radiographic criteria of colonic torsion which could be gleaned when specialized radiographic techniques are not possible.

## Author Contributions

**Nicole Moody:** conceptualization, visualization, writing – original draft, writing – review and editing. **Ashley M. Power:** conceptualization, visualization, writing – original draft, writing – review and editing.

## Funding

The authors have nothing to report.

## Consent

The authors have nothing to report. The dog involved was a privately owned pet and all procedures adhered to ethical guidelines for the respectful treatment of animals.

## Conflicts of Interest

The authors declare no conflicts of interest.

## Data Availability

The authors have nothing to report.

## References

[1] W. A. Endres , D. J. Remondini , and E. R. Graber , “A Case Report of Torsion of the Descending Colon in a Six‐Month‐Old Female Collie,” Veterinary Medicine, Small Animal Clinician 63, no. 10 (1968): 954–960. 5188321

[2] C. A. Carberry and J. A. Flanders , “Cecal‐Colic Volvulus in Two Dogs,” Veterinary Surgery 22, no. 3 (1993): 225–228. 8362506 10.1111/j.1532-950x.1993.tb00387.x

[3] P. M. Shealy and R. A. Henderson , “Canine Intestinal Volvulus. A Report of Nine New Cases,” Veterinary Surgery 21, no. 1 (1992): 15–19. 1580053 10.1111/j.1532-950x.1992.tb00005.x

[4] P. Barge , C. J. Fina , J. R. Mortier , and I. D. Jones , “CT Findings in Five Dogs With Surgically Confirmed Colonic Torsion,” Veterinary Radiology & Ultrasound 61, no. 2 (2020): 190–196, 10.1111/vru.12830. 31837190

[5] E. Davis , F. I. Townsend , J. W. Bennett , J. Takacs , and C. P. Bloch , “Comparison of Surgically Treated Large Versus Small Intestinal Volvulus (2009–2014),” Journal of the American Animal Hospital Association 52, no. 4 (2016): 227–233, 10.5326/JAAHA-MS-6412. 27259023

[6] Z. J. Halfacree , A. L. Beck , K. C. L. Lee , and V. J. Lipscomb , “Torsion and Volvulus of the Transverse and Descending Colon in a German Shepherd Dog,” Journal of Small Animal Practice 47, no. 8 (2006): 468–470, 10.1111/j.1748-5827.2006.00018.x. 16911117

[7] C. S. Park , J. E. Lugardo , C. E. Mans , R. W. Williams , and G. F. Zuendt , “Colonic Torsion and Volvulus in Dogs Is Associated With a Low Mortality Rate and Good Long‐Term Outcome,” Journal of the American Veterinary Medical Association 263, no. 3 (2024): 1–7, 10.2460/javma.24.07.0457. 39366425

[8] P. S. Czajkowski and K. J. Fryer , “Colonic Torsion in 4 Great Danes,” Journal of Veterinary Emergency and Critical Care (San Antonio, TX) 30, no. 5 (2020): 581–586, 10.1111/vec.12986. 32710595

[9] D. Gagnon and B. Brisson , “Predisposing Factors for Colonic Torsion/Volvulus in Dogs: A Retrospective Study of Six Cases (1992–2010),” Journal of the American Animal Hospital Association 49, no. 3 (2013): 169–174, 10.5326/JAAHA-MS-5829. 23535755

[10] S. N. Soulsby , J. M. Balara , and S. N. LeGrange , “What Is Your Diagnosis? Diagnosis: Colonic Volvulus,” Journal of the American Veterinary Medical Association 237, no. 8 (2010): 907–908, 10.2460/javma.237.8.907. 20946076

[11] A. M. Bentley , T. E. O'Toole , M. P. Kowaleski , S. A. Casale , and R. J. McCarthy , “Volvulus of the Colon in Four Dogs,” Journal of the American Veterinary Medical Association 227, no. 2 (2005): 253–256, 10.2460/javma.2005.227.253. 16047662

[12] C. L. Gremillion , M. Savage , and E. B. Cohen , “Radiographic Findings and Clinical Factors in Dogs With Surgically Confirmed or Presumed Colonic Torsion,” Veterinary Radiology & Ultrasound 59, no. 3 (2018): 272–278, 10.1111/vru.12595. 29363214

[13] T. Plavec , S. Rupp , and M. Kessler , “Colonic or Ileocecocolic Volvulus in 13 Dogs (2005–2016),” Veterinary Surgery 46, no. 6 (2017): 851–859, 10.1111/vsu.12674. 28543045

